# Pacbio sequencing of copper-tolerant *Xanthomonas citri* reveals presence of a chimeric plasmid structure and provides insights into reassortment and shuffling of transcription activator-like effectors among *X. citri* strains

**DOI:** 10.1186/s12864-017-4408-9

**Published:** 2018-01-04

**Authors:** Alberto M. Gochez, Jose C. Huguet-Tapia, Gerald V. Minsavage, Deepak Shantaraj, Neha Jalan, Annett Strauß, Thomas Lahaye, Nian Wang, Blanca I. Canteros, Jeffrey B. Jones, Neha Potnis

**Affiliations:** 1 Citrus Pathology, INTA EEA Bella Vista, Bella Vista, Corrientes, Argentina; 2 Department of Plant Pathology, University of Florida, Gainesville, FL USA; 3 Citrus Research and Education Center, Department of Microbiology and Cell Science, IFAS, University of Florida, Lake Alfred, FL USA; 4 University of Tübingen, ZMBP ­ General Genetics, Tuebingen, Germany; 5 Department of Entomology and Plant Pathology, Auburn University, Auburn, AL 36830 USA

**Keywords:** Citrus canker, *pthA*, Pathogenicity, PacBio, Next-generation sequencing NGS, Plasmid co-integration

## Abstract

**Background:**

*Xanthomonas citri*, a causal agent of citrus canker, has been a well-studied model system due to recent availability of whole genome sequences of multiple strains from different geographical regions. Major limitations in our understanding of the evolution of pathogenicity factors in *X. citri* strains sequenced by short-read sequencing methods have been tracking plasmid reshuffling among strains due to inability to accurately assign reads to plasmids, and analyzing repeat regions among strains. *X. citri* harbors major pathogenicity determinants, including variable DNA-binding repeat region containing Transcription Activator-like Effectors (TALEs) on plasmids. The long-read sequencing method, PacBio, has allowed the ability to obtain complete and accurate sequences of TALEs in xanthomonads. We recently sequenced *Xanthomonas citri* str. Xc-03-1638-1-1, a copper tolerant A group strain isolated from grapefruit in 2003 from Argentina using PacBio RS II chemistry. We analyzed plasmid profiles, copy number and location of TALEs in complete genome sequences of *X. citri* strains.

**Results:**

We utilized the power of long reads obtained by PacBio sequencing to enable assembly of a complete genome sequence of strain Xc-03-1638-1-1, including sequences of two plasmids, 249 kb (plasmid harboring copper resistance genes) and 99 kb (pathogenicity plasmid containing TALEs). The pathogenicity plasmid in this strain is a hybrid plasmid containing four TALEs. Due to the intriguing nature of this pathogenicity plasmid with *Tn3*-like transposon association, repetitive elements and multiple putative sites for origins of replication, we might expect alternative structures of this plasmid in nature, illustrating the strong adaptive potential of *X. citri* strains. Analysis of the pathogenicity plasmid among completely sequenced *X. citri* strains, coupled with Southern hybridization of the pathogenicity plasmids, revealed clues to rearrangements of plasmids and resulting reshuffling of TALEs among strains.

**Conclusions:**

We demonstrate in this study the importance of long-read sequencing for obtaining intact sequences of TALEs and plasmids, as well as for identifying rearrangement events including plasmid reshuffling. Rearrangement events, such as the hybrid plasmid in this case, could be a frequent phenomenon in the evolution of *X. citri* strains, although so far it is undetected due to the inability to obtain complete plasmid sequences with short-read sequencing methods.

**Electronic supplementary material:**

The online version of this article (10.1186/s12864-017-4408-9) contains supplementary material, which is available to authorized users.

## Background

*Xanthomonas citri*, the causal agent of citrus canker, represents a well-studied pathosystem due to the detailed understanding of pathogen biology using a combination of approaches including mutational analysis and, more recently, by sequencing hundreds of strains of this pathogen collected around the world. Among various pathogenicity factors deployed by *X. citri* during disease establishment, the most important and widely studied factor includes a type III effector family of Transcriptional Activator-Like effectors (TALEs) [1].

TALEs are DNA-binding proteins that reprogram the expression of specific genes in the host, boosting the expression of susceptibility genes or activating the expression of resistance genes, depending on the host genotype [2]. In the case of *X. citri*, PthA4 is responsible for the elicitation of citrus canker symptoms through the activation of specific host genes, *CsLOB*-1, a member of the lateral organ boundaries (LOB) transcription factor family, and CsSWEET1, a homolog of the SWEET sugar transporter and rice disease susceptibility gene family [1]. Target specificity of TALEs is governed by the central repeat region displaying tandem repeats of a 33–35 aa sequence [3]. Recent structural studies have determined that the number of repeats and the repeat variable di-residues (RVDs) (12th and 13th amino acid) can be used for predicting the target nucleotide sequence that TALEs bind [3, 4]. Thus, the correct determination of the repeat regions of TALEs is important for predicting target sites within the host as well as applications as a tool in genome editing.

The number of repeats present in TALEs varies with an average of 17 repeats and up to >30 repeats [3]. Interestingly, unlike many of the xanthomonads that contain multiple copies of TALEs on the chromosome, *X. citri* strains carry TALEs on plasmids. A model strain for *X. citri*. str. 306 contains four TALEs, with two each residing on two plasmids [5]. TALEs are typically flanked by highly conserved sequences and are often associated with insertion sequence (IS) elements. These features along with repeat-containing sequences make TALE containing regions difficult to assemble accurately. None of the short-read sequencing technologies have been successful in covering the entire sequence of the TALEs, with the read lengths being insufficient to accurately assemble the repeat region. Thus, estimating numbers of repeats as well as copy number of TALEs has been a challenge. In addition, the fragmented regions of TALEs make it difficult to assign it to a particular location in the genome, plasmid or chromosome [6]. Obtaining the intact and accurate sequence and the copy number of TALEs is essential not only to understand pathogen evolution in response to host selection pressure but also to allow identification of host targets.

Variability among *X. citri* strains has been well studied by widespread geographic sampling of the strains from different continents. Gordon et al. [7] conducted comparative analysis of a worldwide collection of 43 strains of *X. citri* belonging to A, A* and A^w^, by sequencing using MiSeq and identified genomic differences including recombination, horizontal gene transfer and single nucleotide polymorphism that could explain differences in host range and virulence of these strains. Since the sequencing platform used in this study yielded draft genomes, authors aligned the obtained reads against the two plasmids, pXAC33 and pXAC64 of *X. citri* str. 306. While such an approach could be sufficient to understand the unique regions associated with host specificity that the authors intended to screen for, a reference based mapping and short-read sequencing approach here might not have captured the rearrangements among the plasmids. In a similar study, Zhang et al. [8] sequenced 21 Asian and North American *X. citri* strains, which belonged to all three A types and identified positive selection as a major factor in evolution of these strains. However, plasmid variation was not considered in their study. In 2005, Carvalho et al. [9] characterized the genetic diversity of 22 *X. citri* A-type strains from South America. Based on the comparison of plasmid profiles, and based on RFLPs identified via pulsed-field-gel electrophoresis, the authors observed high coefficients of similarity for strains isolated in similar geographical locations (from 0.83 to 1 for strains isolated in seven Brazilian states and between 0.62 and 0.83 for strains from Argentina, Bolivia, Paraguay and Uruguay). Also they observed variability in plasmid size (only 5 types of plasmids were observed ranging in size from 57.7 to 83 Kb), indicating plasmid copy number and the size being variable among the strains. In China, Xc-A strains from each of 9 citrus growing regions were characterized in regard to TAL effector variability. As a result, the analysis of 105 strains showed differential pathogenicity for a set of citrus hosts; those strains varied in the number of TAL effectors ranging from 3 to 5 *pthA* genes. Comparison of the strains through DNA restriction using *Bam*HI and hybridization with a probe based on the Xc-A3213 *pthA* gene (*pthA4*) [10], allowed separation of strains into 14 genotypes, with more than 80% of the strains being placed in two major groups according to fragment size. The size difference could be correlated with variation in *pthA* effector sequences identified from other strains [11, 12]. The lack of hybridization observed in some strains indicate the lack of TAL effectors and were correlated with their observed lower virulence [13]. Thus, with TALEs being important virulence factors, differences in virulence of *X. citri* strains on different citrus cultivars may result from the composition of TALEs in strains. A chimeric TALE, *hssB3.0,* was identified as a conserved factor in all tested isolates of the weakly aggressive *X. citri* strains and was characterized as a factor inducing host defense response, partially suppresses canker pathogenesis incited by *pthA,* thus, explaining the role of TALE family members in potentially regulating aggressiveness on a particular host in coordination with other TALEs [14]. In addition, since TALEs are located on plasmids in *X. citri* strains, understanding plasmid variability and shuffling of plasmids among the pathogen population is expected to contribute significantly to our knowledge of pathogen variability and evolution of host range.

Copper resistance (CuR) is commonly observed among *X. citri* strains and has been well studied for the contributing genes for tolerance developed in the strains in recent years. In 1994, Canteros [15] isolated the first CuR strain of *X. citri A* type (Xc-A) from symptomatic lemon trees (cv. Eureka22) in Bella Vista, Corrientes, Argentina. CuR strains of Xc-A have only been isolated in the northeastern region of Argentina. More recently, Xc-A CuR strains were identified in two other provinces in Northeast Argentina (Formosa and Entre Rios) [16]. In 2003, two copper resistant strains were isolated from canker lesions from the same sample of infected grapefruit in the EEA INTA Bella Vista, Argentina, namely, Xc-03-1638 (Xc-A86, also known as LM180, hereafter referred to as LM180) and Xc-03-1638-1-1 (also known as Xc-A44). Molecular characterization of copper resistance operon in strain Xc-03-1638-1-1 (Xc-A44) was carried out recently by Behlau et al. [17]. A different arrangement in the CuR genes was observed between Xc-03-1638-1-1 (*copLABMGCDF*) and *X. citrumelonis* (*copLABMGF*). Both types of *cop* operons, which share conserved nucleotide sequences, have been identified in other copper resistant bacterial species such as *Stenotrophomonas maltophilia* K279a, and *X. vesicatoria* strain 7882 [17]. Copper resistance in *X. citri* strains has been studied worldwide extensively in recent years [18–22]. While the variation in the two strains, LM180, and Xc-03-1638-1-1 was not known at the time of isolation, recent complete genome sequencing of LM180 by Richard et al. [18] and this study indicated that strain level variation can exist at the level of individual samples and such untapped diversity in the composition of plasmids harboring virulence factors can explain adaptive potential of the pathogen.

Our preliminary data indicated the presence of two plasmids in Xc-03-1638-1-1, a bigger plasmid, similar to the size of copper resistant plasmids typically found in xanthomonads and another smaller plasmid. Comparison of the plasmid profile with *X. citri* str. 306, which showed the presence of two plasmids, 33 kb and 66 kb, indicated that the size of the smaller plasmid (~100 kb) from str. Xc-03-1638-1-1 was equivalent to the combination of two plasmids from *X. citri* str. 306. We hypothesized that the 100 kb plasmid in Xc-03-1638-1-1 was a result of plasmid rearrangement. In this study, we have used PacBio sequencing to resolve and obtain complete and accurate sequence and copy number of TALEs and their plasmid location in Xc-03-1638-1-1. We also confirmed our findings using cosmid library construction followed by Sanger sequencing of the clones containing TALEs. We included a strain LM180, which was sequenced earlier by Richard et al. [18]. We compared the two strains for their plasmid content and the whole genome identity. Availability of such simultaneous sampling has allowed us to understand variability exisiting within pathogen populations in a given area.

## Results

### Plasmid profile coupled with Southern hybridization indicates the presence of two larger size plasmids in Xc-03-1638-1-1 compared to reference Xc-A306 strain

**Fig. 1:**
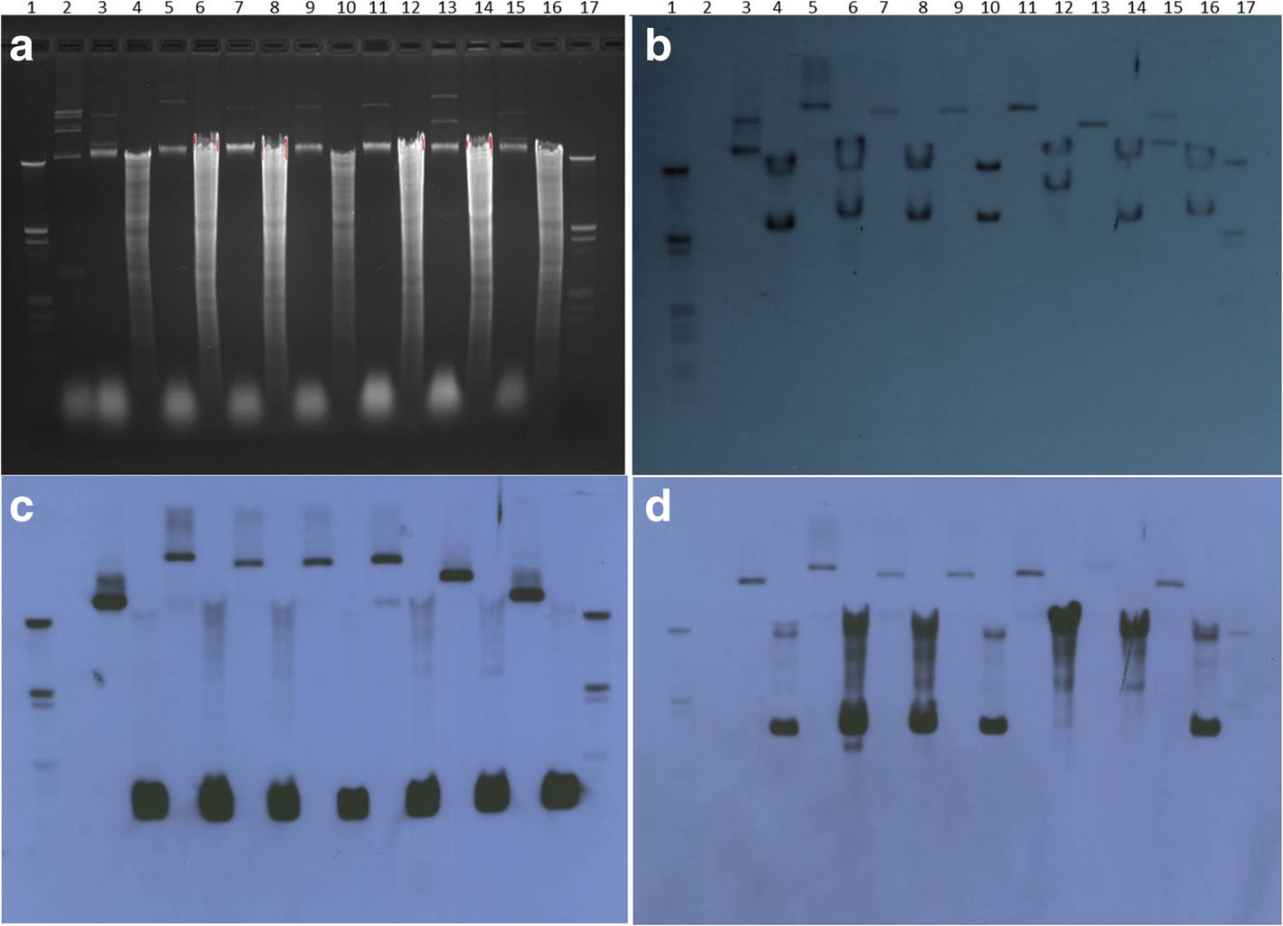
Total genomic and plasmid extractions of strains were subjected to electrophoresis (**a**) and probed with pthA probe (using primer pthAF/R DIG as the probe), Xc-A306-p33 and Xc-A306-p64 plasmid specific probes by Southern hybridization (**b**, **c** and **d**, respectively). Lanes 1 and 17 = DNA Molecular Weight Marker III DIG labeled; 2 = *Erwinia stewartii* SW2; 3 = Xc-A306 plasmid extraction (pXc-A306); 4 = Xc-A306 genomic DNA restricted with EcoRI (Xc-A306 EcoRI); 5 = pXc-03-1638-1-1; 6 = Xc-03-1638-1-1 EcoRI); 7 = pXc-A2090; 8 = Xc-A2090 EcoRI; 9 = pXc-A1660; 10 = Xc-A2090 EcoRI; 11 = pXc-AEtrog; 12 = Xc-AEtrog EcoRI; 13 = pXc-A100-Japan; 14 = Xc-A100 Japan EcoRI; 15 = pXc-A109 India; 16 = Xc-A109 India EcoRI. A = Ethidium bromide stained gel (agarose 0.7%). **b** Southern blot of gel in A with pthA probe. **c** Southern blot of gel in A with Xc-p33 probe (using primer SPCF33F/R DIG as the probe). **d** Southern blot of gel in A with Xc-p64 probe (using primer SPCF64F/R DIG as the probe)

The plasmid profile of Xc-03-1638-1-1 was compared with other *X. citri* strains, XcA2090, XcA1660, XcA-Etrog, XcA100 and XcA109 including Xc-A306 (Table 1), a complete reference genome used in many studies (Fig. 1a). While Xc-A306 contains two plasmids, 33 kb and 64 kb (also confirmed with whole genome sequence) the only strain containing the similar plasmid composition among six *X. citri* A group strains was Xc-A109. Strain XcA03–1638–1-1 contained a larger plasmid band (P1), comparable to the copper resistance plasmid [17] and a second smaller plasmid of size ~100 kb based on the size marker. The size of this second smaller band (P2) was comparable to the size created when the two plasmids from Xc-A306 were combined (Fig. 1a). Three other Xc-A strains contained a single plasmid with ~10 kb smaller size compared to the one from XcA03–1638–1-1. We further designed the probes targeting unique sequences in pXAC33 and pXAC64 from Xc-A306 (Fig. 1c and d) as well as probes targeting TAL effector, *pthA*, (Fig. 1b) to confirm if the single plasmid of size 90Kb-100 kb in the strains XcA03–1638–1-1, Xc-A2090, XcA1660 and XcAEtrog were hybrids of the two plasmids pXAC33 and pXAC64. Southern hybridization using these probes from specific sequences of each of the plasmids from Xc-A306 resulted in the presence of a single band signal (Fig. 1b, c, d) in all of these strains indicating the possibility of recombination of the two plasmids. Further, presence of *pthA* on this single plasmid as indicated by a signal in Southern hybridization (Fig. 1b) indicated that few *X. citri* strains contain a single pathogenicity plasmid, likely the result of plasmid recombination.

**Table 1:** Bacterial strains, plasmid vectors, plasmid constructs and primers used in this study

Designation	Relevant characteristics	Source or reference
*Strains*		
Xc-03-1638-1-1	Red Blush Grapefruit (*C. paradise* Macf.), 2003, Bella Vista, Corrientes, Argentina. CuR	Xcc-03-1638-1-1
Xc-A306	Orange (C. sinensis L. Osbeck) 1997, Paranavai, Parana, Brazil. CuS	Rui Pereira Leite Jr. DPI [5]
Xc-A104	Eureka Lemon (*C. limon* L. Burm. f.), 2008, Bella Vista, Corrientes, Argentina. CuR	Xcc-08-3420
Xc-A79	Clemenule Tangerine (Citrus ×clementina), 2007, Monte Caseros, Entre Rios, Argentina. CuR	Xcc-07-3179
Xc-A86	Red Blush Grapefruit (*C. paradisi* Macf.) 2003, Bella Vista, Corrientes, Argentina. CuR	Xcc03–1638 (LM180) [18]
Xc-A2090	Lime (Citrus sp.), 2004, Monroe County, FL. Lack of pthA1 and pthA4.	DPI
Xc-A1660	Dooryard citrus tree, 2004, Palm Beach County, FL. Idem Xc-2090 DPI	DPI
Xc-AEtrog	Dooryard citrus tree, 2003, Orange County, FL.	DPI
Xc-A100	Satsuma Okitsu (*C. unshiu* Marc.) 1993, Japan). Weak pathogenicity only in KL. No pathogenicity reaction in GF. StrpR	Xcc-93-1900
Xc-A109	Key lime (C. aurantifolia (Christm.) Swingle), 2002, Madras, India. Normal pathogenicity in KL, no pathogenic in GF	Xcc-02-1888
Xv1111	Tomato, 1955, New Zealand. CuR	ATCC 35937.
Xv BV5-4a	Tomato, 1987, Bella Vista, Argentina. CuR	Canteros, B. I.
Xp 1–7	Tomato, 2006, Florida. CuR	Stall, R. E.
SW2	Corn, 1974, Ohio. Erwinia stewartii SW2, 13 mass characterized plasmids.	[42]
*Plasmids*		
pGMTE	pGEMT-easy, AmpR	Promega Corp. (Madison, WI, USA)
pBluescript II SK(+)	Phagemid, pUC derivative, AmpR	Stratagene (La Jolla, CA, USA)
pLAFR3	Tra − Mob+, RK2 replicon, tetR	[45]
*Primers*		
SPCF33–1-F	5’-GCACGTTCTTCTTGGAAGCA-3’	This study
SPCF33–1-R	5’-CCTGGATGAAGTAGTGCAAT-3’	
SPCF64–1-F	5’-CACTCAACGAGTCCCAGCTT-3’	This study
SPCF64–1-R	5’-GTAATCAGGGCGTGCAGGCG-3’	
J-pthA1	5′- CTTCAACTCAAACGCCGGAC-3’	[23]
J-pthA2	5′- CATCGCGCTGTTCGGGAG-3′

### PacBio sequencing assembly yielded a complete genome of X. citri str. Xc-03-1638-1-1 containing two plasmids

Upon identification of unique plasmid composition in Xc-03-1638-1-1, with copper resistance plasmid as well as a possible hybrid pathogenicity plasmid, we sequenced this strain using the PacBio RS II sequencing method (sequencing carried out at ICBR, University of Florida) to dissect the plasmid rearrangements and resulting TALE diversity arising due to plasmid recombination. We obtained a complete gap-free assembly for Xc-03-1638-1-1 strain. PacBio assembly using Canu assembler produced three contigs of 5.19 Mbp, 248 Kbp and 99 Kbp approx. Sizes. The assembled contigs correspond to the predicted genome composition of the strain that contains one chromosome, and two large plasmids, P1 (248kbp) and P2 (99kbp). Chromosome and plasmid 2 were circularized. However, plasmid 1 sequence submitted to GenBank could not be circularized due to lack of overlaps. The average GC composition of the whole genome is 61%. Gene prediction with Prokka indicates that the genome encodes for 4778 CDS and 61 tRNAs (Table 2).

**Table 2:** Genome composition of the strain Xc-03-1638-1-1 that contains one chromosome and two large plasmids obtained after de novo genome assembling process of PacBio SMRT contigs

	Size bp	Predicted CDS	tRNAs	GC content
Chromosome	5,148,873	4387	59	64.74
Plasmid1	249,703	272	2	58.82
Plasmid 2	99,153	148	–	61.29

PacBio assembly of Xc-03-1638-1-1 indicated a 249 kb copper resistance plasmid containing the copper resistance operon flanked by tRNA-Ile. Next to tRNA-Ile lies the IRL sequence of Tn*Xax1*. As indicated previously by Richard et al. [19], the copper resistance operon behaves as a set of passenger genes, and possibly mobilizes between species by intertransposon recombination.

### All four TALEs of the strain Xc-03-1638-1-1 are located on plasmid P2

Our initial observation based on plasmid profile and Southern hybridization using *pthA* probe indicated the presence of TALEs on a single 90 kb/100 kb plasmid in strains Xc-A03–1638–1-1, Xc-A2090, XcA1660 and XcAEtrog (Fig. 1b). We further analyzed PacBio assembly of Xc-03-1638-1-1 for the presence of TALEs. PacBio sequencing allowed us to obtain complete and accurate sequences of TALEs in this strain. Based on PacBio sequencing assembly, four TALEs were determined to be present on a single plasmid P2 (99 kb) (Table 3). To confirm the sequence and Repeat Variable Di-residues (RVDs) of TALEs, we constructed a cosmid library of Xc-03-1638-1-1 to sequence cosmids containing TALEs using Sanger sequencing as an alternative approach. We sequenced cosmid library clones showing positive signals for TALEs using TALE-specific primers (pthAF/R) [1], and also regional specific sequences which identify determined plasmid identities in strain Xc-A306 (SPCF33 and 64 primer sets, Table 1) using Sanger sequencing. The presence of TALEs was identified in several clones of the Xc-03-1638-1-1 library using specific primers for the nuclear localization signal (NLS) located in the terminal part of the *pthA* gene using primers Jpth1/2 [23]. Four cosmid clones, ‘NLS8’, ‘NLS3’, ‘12–5-2’, and ‘5–4-2’, indicated presence of pthA2 and pthA4; pthA1 and pthA2; pthA3; and pthA4 respectively. We obtained 100% identity among the TALE sequences obtained using Sanger sequencing to those obtained using PacBio sequencing. Although we obtained accurate sequences of TALEs, the copy number and location of TALEs could not be confidently identified using cosmid library and Sanger sequencing approach. PacBio sequencing accurately identified the copy number and RVDs of TALEs, which could not be detected using hybrid or individual assemblies using the 454 and Illumina approach (Data not shown). We compared TALE copy number and their location on a single vs multiple plasmids in six other PacBio sequenced *X. citri* strains (Table 3). While we identified three other *X. citri* strains containing three copies of TALEs on a single plasmid, strain Xc-03-1638-1-1 is the only example so far identified with all four TALEs on a single plasmid.

**Table 3:** TALEs and their RVDs from *X. citri* strains compared in this study with their location on respective plasmids

	Plasmid number		Plasmid size	TALE classes			
				Class I (repeat number) (contains *pthA2* and *3* of Xc-A306)	Class II (repeat number) (contains *pthA4* of Xc-A306)	Class III (repeat number) (contains *pthA1* of Xc-A306)	Class IV (repeat number)
Xc-A306	2	Plasmid 1	33,700	15.5		16.5	
		Plasmid 2	64,920	15.5	17.5		
Xc-03-1638-1-1 Argentina	2	Plasmid 1*	249,703				
		Plasmid 2	99,153	15.5 (×2)	17.5	21.5	
LJ207–7 Reunion	3	Plasmid 1*	213,743				
		Plasmid 2	94,139	15.5	17.5	18.5	
		Plasmid 3	49,382			21.5	
LM180 (additional pseudo TALE on plasmid 2) Argentina	4	Plasmid 1*	249,697				
		Plasmid 2	64,978	13.5			
		Plasmid 3	43,340		17.5		
		Plasmid 4	28,949			6.5	
LM199 Argentina	2	Plasmid 1*	197,085				
		Plasmid 2	64,935	15.5			
LH201 Reunion	2	Plasmid 1*	213,737				
		Plasmid 2	92,707	15.5	17.5	21.5	
LH276 Reunion	3	Plasmid 1*	211,337				
		Plasmid 2	73,024	15.5		21.5	
		Plasmid 3	51,192				22.5 (pseudo)
LL074–4 Martinique	2	Plasmid 1*	220,688				
		Plasmid 2	92,708	15.5	17.5	21.5	
		Plasmid 2	47,756				

*Copper resistant plasmid

TALEs were grouped as classes based on their RVD sequences [24]

TALEs analysis and class assignment using AnnoTALEs [24] was used for comparison of TALEs of Xc-03-1638-1-1 to the TALEs from Xc-A306 and the other six PacBio sequenced *X. citri* strains available from NCBI. *X. citri* Xc-03-1638-1-1 contains a variable *pthA1*, with 21.5 repeats (Table 3, Additional file 1: Table S1) compared to *pthA1* of Xc-A306 with 16.5 repeats. Comparison of full-length *pthA1* and RVDs of *pthA1* from six other PacBio sequenced *X. citri* strains indicated that such variable *pthA1* with 21.5 repeats is conserved in four other *X. citri* strains, LJ207–7, LH201, LH276, and LL074–4. TALE analysis grouped *pthA2* and *pthA3* types of Xc-A306 together, both TALE types containing 15.5 repeats. Among all the strains analyzed, only Xc-A306 and Xc-03-1638-1-1 contain TALEs belonging to both subgroups, *pthA2* and *pthA3*. Other strains LH276, LJ207–7, LH201, LM180, LM199 contain only a single copy from this group, either *pthA2* or *pthA3*. Upon alignment of RVDs of TALEs from this group, we subgrouped the TALEs within this group as *pthA2* type and *pthA3* type (according to Xc-A306 classification). Interestingly, strain Xc-03-1638-1-1 *pthA2* shares the last two RVDs of the *pthA3* of Xc-A306. TALE1 in Xc-03-1638-1-1 which belongs to *pthA3* subgroup differs from *pthA3* of Xc-A306 at 2 amino acid positions that are part of the RVDs. On the other hand, TALE2, that belongs to the *pthA2* subgroup of Xc-A306, shares 100% identity with the N terminal portion and RVD region of *pthA3* of Xc-A306 (Additional file 1: Table S1). Such a hybrid variant of the *pthA2* subgroup TALE from Xc-03-1638-1-1 could have resulted during plasmid co-integration.

### A large P2 plasmid with all four TALEs shows evidence of rearrangement as a result of Tn*3*-mediated transposition and plasmid co-integration

**Fig. 2:**
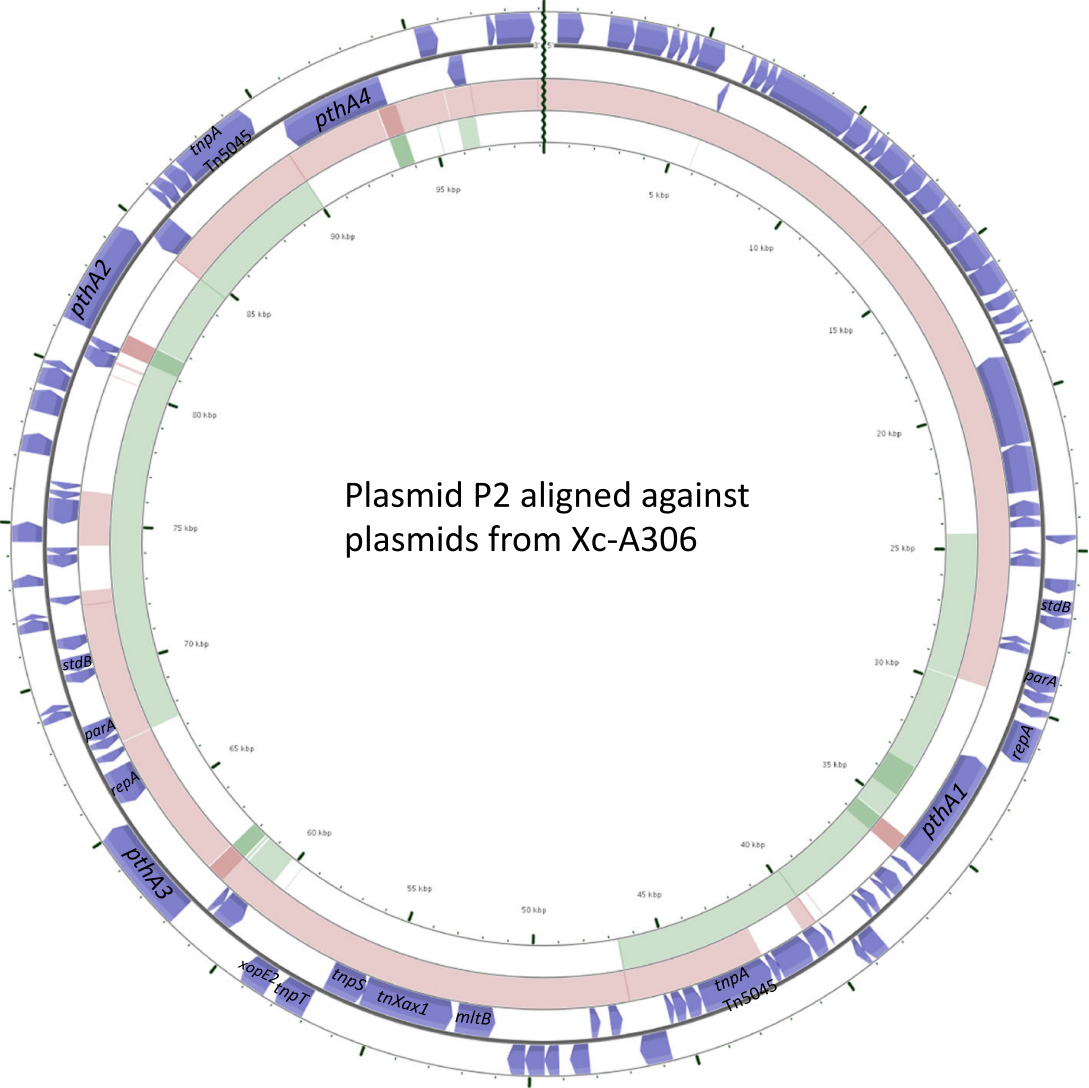
A composite plasmid genome comparison figure was generated using CGView comparison tool after performing a BLASTN analysis of plasmid 2 of Xc-03-1638-1-1 against pXAC33 and pXAC64 plasmids of *X. citri* str. 306. Each plasmid genome mapping to plasmid 2 is represented as a colored ring with a solid color representing greater than 99% sequence identity and the lighter color showing 99% sequence identity. The TALEs, transposases and genes involved in replication and stability are labeled. The outer two rings indicate ORFs encoded in plasmid P2 of Xc-03-1638-1-1 (annotated using prokka). The inner two rings, with brown ring representing pXAC64 and green ring representing pXAC33 plasmids, are aligned against plasmid P2 of Xc-03-1638-1-1 using 99% sequence identity and E-value of 0.001 as a cut-off

**Fig. 3:**
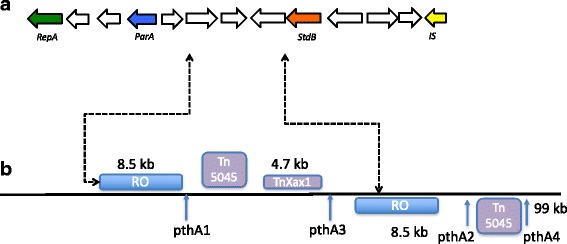
**a** Figure shows the detailed organization of the origins of replication in plasmid P2 . Arrows in color indicate genes coding for proteins associated with plasmid replication and maintenance. In green Replication RepA protein; Blue, Partitioning ParA protein; Orange, Stability StdB protein. In yellow putative insertion sequence. Arrows in white code for hypothetical proteins. **b** The figure shows the position of origin of replication repeats (RO) in plasmid P2. The figure also shows the position of the repeated Tn*3*-like element (Tn*3*) Tn*Xax1* and Tn*5045* as well as positions of TALEs

Ferreira et al. [25] described the Tn*Xax1* family of Tn-3 like transposons that form mobile insertion cassettes (MICs). Tn*3*-like elements are replicative transposons that during a transposition event conduct temporary co-integration of donor and target molecules with a later resolution to copy the transposable element. Their study also described TALEs being passenger genes of MICs and characterized an 8944 bp canonical Tn*Xax1* located on plasmid pXAC64 of Xc-A306. We screened the Xc-03-1638-1-1 genome and plasmids for inverted repeats associated with Tn*Xax1* and Tn*Xax1* sequences (Fig. 2). Plasmid P2 contains 8944 bp Tn*Xax1* canonical region located between coordinates 50,655 and 59,599. Tn*Xax1* right inverted repeat (IRR) was located between 50,655–50,746. The two sites for Tn*Xax1* left inverted repeat (IRL) were identified in plasmid P2, and located between 59,599–59,509 and 95,237–95,327. The 8944 bp canonical Tn*Xax1* of plasmid 2 is conserved similar to the one observed in pXAC64 containing *tnpA* encoding transposase of Tn*5044* family with DDE motif. A functional resolution recombination site, Tn3 family *rst* site, present in the intergenic region flanking *tnpS* and *tnpT*, contains palindromes, one pair at which Tnp*T* binds and the other site is a part of the core site at which recombination takes place. This Tn*3* family rst site is located between 56,850–57,040 of plasmid P2, with palindromic sequences. Plasmid P2 contains two identical copies of Tn*5045* transposase located between 41,850–44,297 and 87,083–89,632 that also belong to Tn*3* family that are present in pXAC64 and pXAC33 plasmids. Tn*5045* shares 76% nucleotide identity with the transposon Tn*5044*, Tn*Xax1* (described above). In *X. citri* str. 306, *pthA2* and *pthA4* are in close proximity of Tn*5045* transposase; while in Xc-03-1638-1-1, Tn*5045* transposase contains *pthA1* (near one copy) and *pthA2* and *pthA4* (in close proximity to the other copy) (Figs. 2 and 3). The Tn*3*-like system resembles bacterial IS1 elements that have been shown to catalyze plasmid cointegration [26]. Inverted repeats containing a potential core recombination site in this region could explain the chimeric structure of plasmid P2 derived by recombination between Tn*Xax1* from pXAC64 and pXAC33 or other closely related A strains containing a Tn*Xax1* copy with a *pthA1* variant.

We identified four direct repeats regions of 1 to 1.6 Kb and three inverted repeats of 8.5 Kb, 4.7 Kb and 1 Kb in plasmid P2 (Table 4). Interestingly the two 8567 bp repeats located in plasmid P2 at positions 24,407 and 72,763 encode for proteins associated with replication and maintenance (Fig. 3). Thirteen coding sequences (CDSs) are predicted in this duplicate region. The first CDS is predicted to encode for a 406 aa protein with 100% identity to the replication protein A in plasmid *X. citri* str. 306 (Genbank sequence sequence ID WP001052942). The fourth and ninth CDSs encode for products of 208 aa and 165 aa with identity of 100% with partition and stability proteins respectively (Genbank sequence ID WP005931726 and WP080767064). It is suggestive that these regions are remnant of plasmids that recombined and integrated to produce a larger replicon.

**Table 4:** Repeat regions in plasmid P2 of strain Xc-03-1638-1-1

Start1	Start2	Length	Orientation
35,080	92,950	1054	F
64,194	91,242	1072	R
60,207	74,976	1196	F
41,882	89,498	4785^a^	R
24,407	72,763	8567^b^	R
31,905	90,171	1069	F
61,615	81,098	1655	F

^a^Tn3-like transposon

^b^Replication origin

### Whole genome comparisons of two strains isolated from the same infected sample indicated adaptive potential of *X. citri* strains bearing plasmids with differences in TALEs repertoires with nearly identical conserved chromosomes

**Fig. 4:**
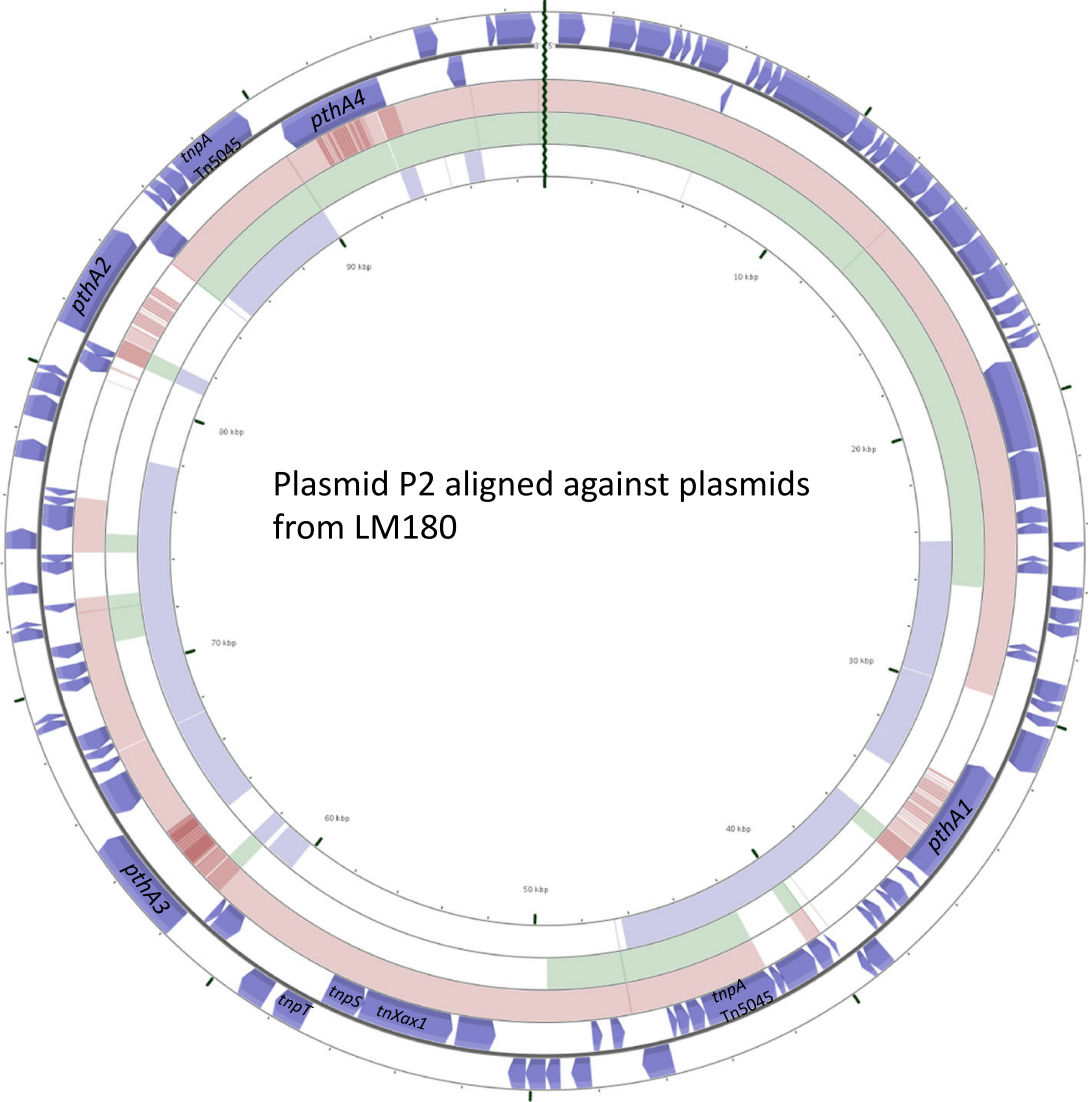
A composite plasmid genome comparison figure was generated using CGView comparison tool after performing a BLASTN analysis of plasmid 2 of Xc-03-1638-1-1 against LM180.2, pLM180.3 and pLM180.4 plasmids of *X. citri* str. LM180, isolated from same infected grapefruit sample as Xc-03-1638-1-1. Each plasmid genome mapping to plasmid 2 is represented as a colored ring with a solid color representing greater than 99% sequence identity and the lighter color showing 99% sequence identity. The TALEs, and transposases are labeled. The outer two rings indicate ORFs encoded in plasmid P2 of Xc-03-1638-1-1 (annotated using prokka, dark purple color). The inner three rings, with brown ring representing pLM180.2, green ring representing pLM180.3 and light purple ring representing pLM180.4 plasmids, are aligned against plasmid P2 of Xc-03-1638-1-1 using 99% sequence identity and E-value of 0.001 as a cut-off

We compared whole genome identities between Xc-03-1638-1-1 and LM180 along with other sequenced genomes in this study. ANI indices indicated nearly identical chromosomes from all compared *X. citri* genomes in this study. The chromosomes of the two strains differed by 52 SNPs with near-identical chromosomes based on Mauve alignment (Additional file 2: Table S2, Additional file 3: Figure S1). It is interesting to note that Xc-03-1638-1-1 and LM180, two strains obtained from the same infected grapefruit leaf sample, although sharing a copper resistant plasmid, differed remarkably in the plasmid copy number as well as TALE composition (Table 3). We compared plasmid P2 of Xc-03-1638-1-1 to the three plasmids of LM180, pLM180.2, pLM180.3, and pLM180.4, using CGView comparison tool [27] (Fig. 4). Plasmid pLM180.2, (64 kb in size) contains one intact TALE with 13.5 repeats (belonging to *pthA2/3* subgroup, Additional file 1: Table S1). Plasmid pLM180.3 and pLM180.4, both of which contain one TALE, *pthA4* homolog (17.5 repeats, with one RVD sequence different than *pthA4* from Xc-03-1638-1-1) and TALE with 6.5 repeats (belonging to *pthA1* subgroup) respectively (Additional file 1: Table S1). Plasmid pLM180.2 contains 8944 bp Tn*Xax1* canonical region, with the resolution recombination site. Interestingly, both pLM180.2 and pLM180.3 contain type IV secretion system genes. Plasmid pLM180.4 contains two copies of 8.5 kb RO region, containing replication origin protein (encoded by *repA*), plasmid partitioning protein (encoded by *parA*) and stabilization protein (encoded by *stdB*), that are also found in plasmid P2 of Xc-03-1638-1-1. These observations indicate that multiple forms of plasmid(s) with differential gene repertoires exist during a single season in a single field. Such a finding is similar to the one observed by Canteros et al. [28], where the authors showed that at least 8 plasmid size classes could be transferred to a marked strain during a single growing season.

## Discussion

In this study, we have utilized the power of long-read PacBio sequencing technology to obtain complete accurate assembly of complete genome of *X. citri* strain including accurate assembly of plasmids. We chose the strain Xc-03-1638-1-1 for PacBio sequencing given the presence of a unique plasmid profile as well as presence of a unique TALE, PthA1 containing 21.5 repeats based on our preliminary studies [14, 29]. We accurately assembled TALEs, determined the assignment of TALEs to the plasmids and identified plasmid rearrangements in this PacBio sequenced *X. citri* strain infecting grapefruit. In *X. citri* pathosystem, TALE, more specifically, PthA4, has been characterized as a major virulence factor, with the corresponding susceptibility factor, being, CsLOB-1 [1]. These and other studies in other *Xanthomonas* pathosystems [2–4] have highlighted the importance of obtaining complete sequences of RVDs of TALEs to decipher target specificities. A complete genome sequence of *X. citri* str. 306 available in 2002 indicated the presence of two TALEs each on two plasmids. The genome sequencing efforts over the next decade included utilization of short-read sequencing methods. A majority of studies involving changes at the genomic level in *X. citri* populations have utilized draft genome sequences, which have provided insights into the variation within strains at the level of SNPs or presence/absence of individual gene/s or clusters. Major shortcomings using short-read sequencing methods to study pathogen variation have been difficult in assigning contig sequences to plasmids, identifying accurate numbers of plasmids and assembling repeat regions, specifically, TALEs, accurately. Such shortcomings have been overcome by long-read PacBio sequencing technology. Both plasmids as well as repeat-containing regions belong to the pool of mobile genetic elements. Such elements are important contributors to the pathogen variation, offering fitness advantage to the pathogen due to their ability to transfer pathogenicity factors. Repeat-containing virulence factors, TALEs, are major pathogenicity factors of *X. citri* and have been well studied for their role in programming the host in the benefit of the pathogen. The Pacbio sequencing method has resolved TALEs repeat sequences successfully in many xanthomonads [30, 31]. In this study, PacBio sequencing of *X. citri* strain Xc-03-1638-1-1 indicated the presence of four copies of TALEs on a single plasmid. We also analyzed TALEs along with their copy numbers for their presence on plasmids of other available PacBio sequenced *X. citri* strains obtained from NCBI GenBank. Interestingly, Xc-03-1638-1-1 is the only *X. citri* strain containing all four copies on a single plasmid. Strain Xc-03-1638, also known as LM180, isolated simultaneously from the same infected grapefruit sample, displayed a very different TALE composition along with their presence on multiple plasmids, although average nucleotide identity at the chromosomal level was over 99%. Interestingly, LM180 contains three plasmids, each containing one TALE. Plasmid pLM180.2 (64 kb in size) contains the canonical Tn*Xax1* region, possibly involved in plasmid co-integration. Among repeat regions screened among the three plasmids, Tn*5045* spanning region is present in all three plasmids, while two sites spanning origin of replication region were identified in plasmid pLM180.4. This particular example indicates the extent to which plasmid variation could exist in a single infected sample along with multiple variants of TALEs available as a part of variable gene pool. We also noted that none of the TALEs from Xc-03-1638-1-1 were 100% identical to the TALEs from simultaneously isolated LM180. PthA4 was found in both strains, with difference in one RVD sequence. LM180 contained differences in repeat numbers fro *pthA2/3* subgroup and contained *pthA1* version with 6.5 repeats. Thus, multiple forms of TALEs, with different repeat numbers and RVD sequences were identified in a single infected sample. Such multiple plasmid variants present simultaneously during infection might impart adaptive plasticity to the pathogen.

The variable plasmid configuration, that we observed in Xc-A strains in this study, was also previously reported by Amuthan & Mahadeban [32], and Carvalho et al. [9]. Recently, it was determined in specific strains of *Pseudomonas syringae* pv. *phaseolicola* [33] and *X. a.* pv. *malvacearum* [34], that *in planta* effects induce changes in its plasmid profile structure, through loss and rearrangement of plasmids after infiltration, or exposure of the pathogen to host leaf extracts. In *P. s*. pv. *syringae* (Pss) a possible explanation for the diversity in size of copper resistance plasmids observed in several isolates was proposed after the demonstration of the co-integration of Pss-pPT23D (35 Kpb; allows CuR), with Pssp-PS6 (a plasmid present in CuS strains). The obtained CuR tranconjugants showed plasmids of 60 Kbp and 100 Kpb size, similar to the ones present in CuR strains of *X. vesicatoria* previously isolated [35]. In this study, some of the strains, that we compared, share plasmids pXAC33, pXAC64, and P2 size configurations, and even dissimilar profiles which did not match any of the other configurations described above. Thus, availability of complete genome sequences of *X. citri* strains have allowed us to understand the extent of variation in terms of rearrangements of genomic fragments or plasmids. The degree of heterogeneity of the pathogen population, governed by the variable portion of the genome, which includes plasmids carrying virulence factors, is an important consideration for understanding how host selection pressure is effective against pathogen population in a given area and can have consequences in designing breeding programs.

Ferreira et al. [25] emphasized the importance of *Tn3*-like transposon elements in reassortment of TALEs among *X. citri* strains on single or multiple plasmids proposing a model of variability arising in the number of TALE gene repeats as a result of *Tn3* family replicative transposition. Such *Tn3*-like transposons have also been proposed to be responsible for mobilizing passenger genes, which include virulence genes, thus, potentially mediating the spread of the different combinations of virulence factors as well as their diversification. Such integron-like mechanism of reassortment of TALEs in clusters has been described in *X. oryzae pv. oryzicola* [36]. Strain Xc-03-1638-1-1 is a perfect example of how *Tn3*-like transposons have contributed to the plasmid co-integration and resulting combinations of TALEs. The P2 pathogenicity-associated plasmid contains four TALEs necessary to produce typical citrus canker symptoms. By comparison with the reference genome of strain Xc-A306, the sequence of P2 could be explained by a recombination event between plasmid pXAC33 and pXAC64 found in Xc-A306. Presence of multiple *Tn3*-like elements in plasmid P2 sequence could lead to differential rearrangement with a different number of *pthA* genes as a result of recombination. As was described in *X. a.* pv. *glycines*, the presence of regions rich in transposons allows the possibility for recombination between plasmids, where several plasmid variants which are based on a non-described plasmid prototype, share transposase, integrase and resolvase genes [37]. The presence of *Tn3*-like transposition could be a mechanism for generating variants in plasmids, especially pathogenicity plasmids carrying variants of TALEs in *X. citri*. Interestingly such *Tn3*-mediated transposition could also have been responsible for changes in repeat numbers, RVDs due to replication slippage during the replicative stage of transposition or by unequal crossing-over [25]. Interestingly, we also observed a chimeric *pthA2* subgroup in Xc-03-1638-1-1, that shared 100% identity with the N terminal portion and RVD region of *pthA3* of Xc-A306. Such modular recombination of TALEs could have resulted during a transposition event. The presence of Xc-A strains with different numbers of TALEs was reported by Lin et at. [11, 38] where they found strains with 3 to 6 *pthA* homologs genes. It is possible to speculate that the origin of plasmid P2 is related to co-integration of older plasmids and these events have been driven by *Tn3*-like transposable elements and incomplete events of resolution.

Plasmid P2 represents an intriguing structure due its repeated elements, and also the number of putative sites for origin of replication. These observations open questions with regard to its stability. We cannot rule out the possibility that alternative structures of plasmid P2 co-exists as autonomous replicons in strain Xc-03-1638-1-1, especially given the fact that two copies of 8.5 kb regions containing genes involved in plasmid replication, partitioning and stability are present. Repeats within the plasmid can cause rearrangements in co-integrates. The origin of replication in resolved structures might allow their stability and maintenance. Previous observations based on analysis of cosmid clones indicate that Xc-03-1638-1-1 might contain 5 TALEs. This could be explained by the possibility of rearrangements and resolution of co-integrates of the plasmid P2. To rule out this possibility, we analyzed in detail the non-used Pacbio reads in the Plasmid assembly (reads that do not form part of the consensus) and ran Blastn against the Plasmid. We found few reads that still align with the consensus and show minor discrepancies (reason those were not included). We could find only a minor set of larges reads (four) that aligned partially and show with major rearrangements. These reads might represent two possibilities. These could be bona fide recombination events that were captured during the sequencing process (sequencing in real time) or we cannot rule out the possibility of possible artifacts during the library preparation. In our case, the small number of these reads precludes a conclusive sentence about possible rearrangements of plasmid 2, but opens the possibility of using higher coverage to rescue putative recombination events that produce rearrangements in the Xc-03-1638-1-1 genome.

The presence of events such as plasmid co-integration, mediated by transposases, as in this case, could be one of the mechanisms to create multiple possible combinations of carrier genes and could play an essential role in adaptive plasticity. PacBio sequencing of Xc-03-1638-1-1 and comparison with other completely sequenced *X. citri* genomes indicated evidence of recombination events in *X. citri* strains that might have been responsible for frequent shuffling of TALEs among strains. TALEs, being major pathogenicity determinants in *X. citri* strains, are subject to selection pressure in the host. Evolution of TALEs in terms of RVDs and copy numbers is an important consideration to understand pathogen population structure. Plasmid co-integration with all four TALEs on a single plasmid of Xc-03-1638-1-1 could be an important strategy of the pathogen to mobilize a pathogenicity plasmid to ensure fitness. Recent study with *X. oryzae pv. oryzae* and *X. oryzae pv. oryzicola* TALEs also indicated that TALEs have evolved via a number of modifications including base substitutions in codons of RVDs, repeat number variability or by recombination [30]. Furthermore, this study also indicated integron-like mechanism responsible for reassortment of TALEs. It is also important to consider the genome plasticity of this pathogen that is offered by *Tn3*-like transposition events that allows the pathogen to be able to acquire novel variants of TALEs or delete a TALE version that is recognized by the host. Such a phenomenon of plasmid recombination and reshuffling of TALEs might have been a common phenomenon that has not been investigated given the majority of *X. citri* genomes being sequenced by short-read sequencing methods. Such evolutionary modifications including presence/absence of TALEs, RVD swaps, chimeric forms of TALEs or pseudogenization of TALEs could lead to differential recognition specificities of the target host genes. Thus, Tn3-like transposons have shaped the evolution of *X. citri* strains by offering plasticity to the genome by plasmid co-integration events, thereby, modifying TALE copies, generating new chimeric TALE variants, as well as by integration of plasmid elements into the chromosome. Such genome plasticity could be an important strategy of *X. citri* strains while adapting to a new ecological niche or as a modification in response to the host selection pressure.

## Conclusions

In this study, we utilized long-read sequencing technology to study a previously unexplored area of pathogen biology, namely, plasmid variation. While a number of studies have recently explored variation in TALEs and their significance in host resistance/susceptibility, this study demonstrates that plasmid reshuffling events could play a role in maintaining variable TALE copies as well as generating novel variants of TALEs with variable RVDs sequences or repeats in *X. citri* strains. Availability of whole genome sequences of the two strains simultaneously isolated from the same infected leaf sample has provided clues as to the variability in plasmid copy number as well as associated virulence factors that can exist. These findings indicate that maintaining several forms of plasmids that contain variable copies of virulence factors, at a time in the pathogen population could be a strategy that the pathogen can utilize to adapt to host selection pressure.

## Methods

### Bacterial strains and media

Strains utilized in this work are listed in Table 1. Strain Xc-03-1638-1-1 was stored in glycerol stock at -80 °C and was subcultured on nutrient agar (NA) medium. Rifampicin resistant strains were obtained by plating 10^9^ colony-forming-units (CFU)/ml on NA containing 50 μg/ml of rifampicin. Xc-03-1638-1-1 and *Escherichia coli* (Ec) strains were maintained on Luria-Bertani (LB) medium [39], and also stored in nutrient broth (Difco™) containing 30% glycerol, in a − 80 °C freezer.

### Isolation of plasmid DNA

Bacterial strains were grown overnight in 4 mL nutrient broth (NB) at 28 °C under agitation at 250 rpm using a KS10 orbital shaker (BEA-Enprotech Corp., Hyde Park, MA). Bacterial cell suspensions were then standardized to an OD of 0.3 A at 600 nm using a spectrophotometer. Plasmid DNA was extracted following the method of Kado and Liu [40] with modifications [41]. Detection of plasmids was performed by electrophoresis as described previously [41]. After extraction, 28 μL of individual plasmid preparations were run in a 0.5% agarose gel, stained with ethidium bromide (0.5 μg mL-1) for 30 min and photographed using a UV transilluminator and Quantity One software (Bio-Rad Universal Hood II, Hercules, CA). Plasmids of *Pantoea stewartii* SW2 (syn. *Erwinia stewartii* see Table 1) were used as molecular size markers [42].

Southern hybridization experiments were performed on positively charged nylon membranes and the DIG-High Prime DNA Labeling and detection kit, according to the manufacturer’s instructions (Roche). Genomic DNA extractions were made using the CTAB- DNA isolation method [39]. DNA preparations were digested with *Eco*RI endonuclease (Promega). The digested DNA was electrophoresed in 0.8% agarose gel, using as a size reference marker DIG labeled 0.12–21.2 Kbp DNA Molecular Weight Marker III (Roche).

### PCR analysis

Primers were synthesized by Sigma-Aldrich (Sigma-Aldrich Co., St. Louis, MO). Amplification of target genes from all bacteria was performed using a DNA thermal cycler (Bio-Rad-MyCycler™) and the Taq polymerase kit (Promega, Madison, WI). For extraction of template DNA, strains were individually grown overnight on NA, suspended in sterile deionized water (DI), boiled for 20 min, cooled on ice for 5 min, shaken thoroughly using a vortex mixer (Genie2, Scientific Indusctries Inc., NY), centrifuged at 15,000 rpm for 5 min and placed on ice until the supernatant was used in the PCR reaction mixture. Each PCR reaction mixture, prepared in 25 μL total volume, consisted of 11.4 μL of sterile water, 5 μL of 5 × PCR buffer, 1.5 μL of 25 mM MgCl2, 4 μL deoxyribonucleoside triphosphates (0.8 mM each dATP, dTTP, dGTP, and dCTP), 0.5 μL of each primer (stock concentration, 25 pmol μL-1), 2 μL of template, and 0.2 μL (5 U/μl) of Taq DNA polymerase. PCR reactions were initially incubated at 95 °C for 5 min. This was followed by 30 PCR cycles which were run under the following conditions: denaturation at 95 °C for 30 s, primer annealing for 30 s at 5 °C below the minimum primer Tm, calculated for each primer based on the following formula: Tm = [81.5 + (41*(%GC/100))-21.6-(500/(length)], and DNA extension at 72 °C for 45 s in each cycle. After the last cycle, PCR tubes were incubated for 10 min at 72 °C and then placed at 4 °C. In every reaction, a positive control was included depending on the set of primers. PCR reaction mixtures were analyzed by 1% agarose gel electrophoresis (Bio-Rad Laboratories, Hercules, CA) with Tris-acetate-EDTA (TAE) buffer system. A λ-*Eco*RI-*Hind*III DNA marker (Promega, Madison, WI) was used as the standard molecular size marker for PCR product sizing. Reaction products were visualized by staining the gel with ethidium bromide (0.5 μg mL-1) for 20 min and then photographed using a UV transilluminator and Quantity One software (Bio-Rad Universal Hood II, Hercules, CA).

### Cosmid cloning

The genomic library of Xc-03-1638-1-1 was constructed in the vector, pLAFR-3 [43–46], following digestion with the restriction enzyme *Sau3*AI (NEB). Individual clones were maintained in Ec DH5α (BRL). Subcloning, plasmid alkaline lysis, and agarose gel electrophoresis were essentially as described by Sambrook et al. [39]. Specific clones from the Xc-03-1638-1-1 library were digested with restriction enzymes, religated, and sequenced using the Sanger method (ICBR, UF).

### PacBio RS II sequencing

DNA sample for Xc-03-1638-1-1 was sequenced using PacBio single molecule real-time (SMRT) technology. Two SMRT libraries were sequenced using P5-C3 chemistry generating 41,382 reads with and average length of 6.6 Kb yielding ~55X coverage of the predicted genome. De novo assembly was conducted using hierarchical assembly pipeline (HGAP) implemented in Canu V1.3 software [47]. Raw PacBio reads were mapped against the resulting contigs using blasR aligner and SNPs corrections were conducted with variant-caller software using the quiver algorithm. Genome gene prediction and annotation was conducted using Prokka annotation pipeline [48] as well as by IMG-JGI annotation pipeline. Genome sequences have been assigned to accession number CP023285-CP023287 under BioProject PRJNA401937, BioSample SAMN07611881. Pacbio raw sequence data files (bas.h5 and bax.h5) are available at NCBI’s Sequence Read Archive (SRA) database under the accession number SRP126789.

## Additional files

Additional file 1: Table S1. Repeat Variable Di-residues (RVDs) of different groups of TALEs from *X. citri* strains sequenced using pacbio sequencing strategy including Xc-03-1638-1-1. The TALEs have been grouped in three types, pthA2/3 type, with two subgroups as pthA2 and pthA3; pthA4 type and pthA1 type. Note the residues highlighted for TALEs in Xc-03-1638-1-1 show chimeric version of pthA2/3 type upon comparing RVDs. (XLS 27 kb)

Additional file 2: Table S2. List of SNPs obtained upon comparing chromosomes of Xc-03-1638-1-1 and LM180. (XLS 44 kb)

Additional file 3: Figure S1. Whole genome alignment generated by Mauve software to show chromosomal conservation among Xc-03-1638-1-1 and XcA306. Genome similarity/conservation among these two strains is evident based on height of bars. (JPEG 51 kb)

## Abbreviations

TALE: Transcription Activator-Like Effectors
Xc-A: *Xanthomonas citri A* type
