# Supplementary figures and images for: Pacbio sequencing of copper-tolerant Xanthomonas citri reveals presence of a chimeric plasmid structure and provides insights into reassortment and shuffling of transcription activator-like effectors among X. citri strains
Source: BMC Genomics. 2018 Jan 4;19:16. doi: 10.1186/s12864-017-4408-9 (PMC5755412; doi:10.1186/s12864-017-4408-9)

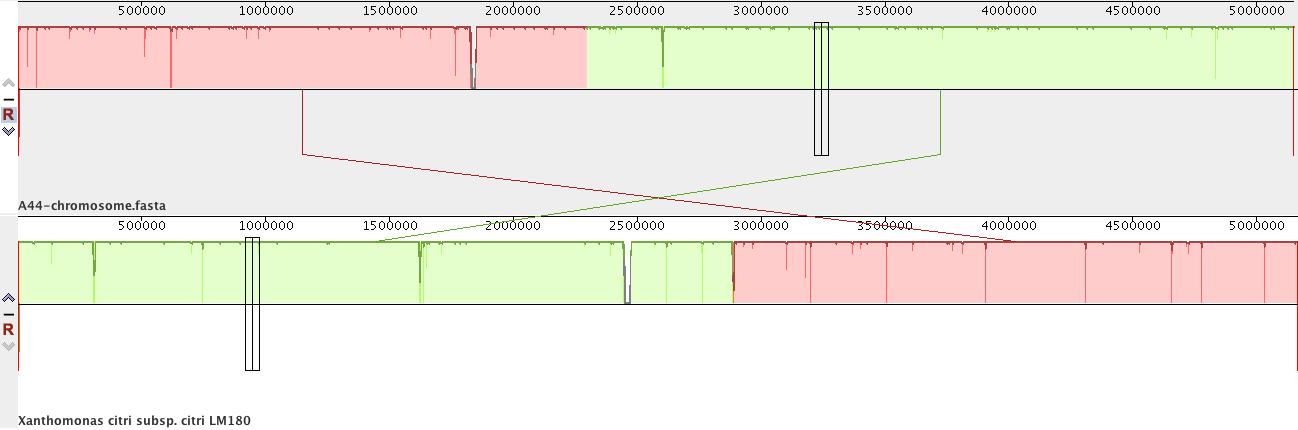

Supplement: Supplementary file 3 — Whole genome alignment generated by Mauve software to show chromosomal conservation among Xc-03-1638-1-1 and XcA306. Genome similarity/conservation among these two strains is evident based on height of bars. (JPEG 51 kb) [file 12864_2017_4408_MOESM3_ESM.jpg]
